# Baby names in Japan, 2004–2018: common writings and their readings

**DOI:** 10.1186/s13104-020-05409-3

**Published:** 2020-12-08

**Authors:** Yuji Ogihara

**Affiliations:** Faculty of Science Division II, Tokyo University of Science, Kagurazaka, Shinjuku-ku, Tokyo 162-8601 Japan

**Keywords:** Name, Baby, Writing, Reading, Uniqueness, Individualism, Naming, Open data, Japan, Chinese character

## Abstract

**Objectives:**

To conduct empirical research on Japanese names, actual name data including both writings and readings are necessary. However, there was no database available that met these conditions. Therefore, in the present article, I provided raw data of approximately 8000 names of Japanese babies born between 2004 and 2018.

**Data description:**

The data include common writings of baby names and their readings generated from annual surveys on baby names conducted by a Japanese private company. The data have advantages: (1) they include both writings and readings of baby names, (2) they were collected under the same conditions over 15 years, (3) their sample sizes are relatively large, and (4) they are open to the public. In contrast, the data have limitations: their samples are neither highly representative nor very large. Overall, this article will be useful for empirical research on Japanese names and people in general (especially for medical and educational service workers).

## Objective

Names affect us in social, economic, psychological, and other ways. Thus, names have been studied in interdisciplinary fields. For example, sociological and psychological research has demonstrated that unique names have increased over time in Germany [1], the U.S. [2, 3], Japan [4, 5] and China ([6]; but also see [7]). This phenomenon shows that people increasingly seek more uniqueness, indicating the rise of individualism [8, 9].

### Name data in Japan

To conduct empirical research on names, actual name data (not hypothetical or anecdotal names) are necessary. Moreover, raw data including both writings and readings of names are required in Japan [10].

However, there is no database available that includes both writings and readings of baby names, at least currently in Japan. Although some private companies have published rankings of the most common writings and/or readings of baby names, these rankings are separate, and data on names with both their writings and readings are lacking.

Therefore, to fill this gap, the present article reports raw data on recent baby names with both writings and readings in Japan. I collected baby names from open data published by a private company in Japan.

### Practical implications

Raw data of baby names in Japan are important not only for researchers but also for people in general. The data show many actual examples of names and illuminate recent trends in baby names. Moreover, the data include both common and unique names in Japan.

These data are valuable, particularly for medical service workers (e.g., doctors, nurses, and hospital staff) and educational service workers (e.g., teachers, instructors, and school staff). This is because it is important for these professionals to be able to read names correctly. They want to avoid situations where they have no idea of how to read names and make mistakes in doing so.

## Data description

### Data

The data of the present report were collected from annual surveys on baby names conducted by the Meiji Yasuda Life Insurance Company [11]. Meiji Yasuda has collected baby names from their customers who have babies each year to understand trends in baby names.

Although Meiji Yasuda has not published raw data of baby names, it has published part of its survey results online annually since 2004. These results include three types of rankings and two types of lists. The three types of rankings are those of the most common writings, readings, and Chinese characters. The two types of lists are for readings of the top 10 most common writings and for writings of the top three most common readings.

In this report, to collect raw data of baby names, I used the lists for the readings of the top 10 most common writings for the 15 years between 2004 and 2018. The data of the present report are publicly available [12]. The data include writings, readings (in alphabet and hiragana), number of babies with each name, and their percentages among each sample.

### Sample size

The sample sizes are summarized in Table S1 (see Table 1). The total sample sizes for the present data were 3762 for boys and 4017 for girls (a total of 7779 names). The average annual sample sizes were 251 for boys and 268 for girls.

**Table 1:** Overview of data files

Label	Name of data file/data set	File types (file extension)	Data repository and identifier (DOI or accession number)
Data file 1	Data Baby names Japan	Microsoft Excel file (.xlsx)	Open Science Framework (10.17605/OSF.IO/2WURJ)
Data file 2	Table S1 Sample sizes for present data and original surveys	Microsoft Word file (.docx)	Open Science Framework (10.17605/OSF.IO/2WURJ)
Data file 3	Table S2 Numbers of variations and Hiragana names	Microsoft Word file (.docx)	Open Science Framework (10.17605/OSF.IO/2WURJ)
Data file 4	Supplementary material Advantages of the present data	Microsoft Word file (.docx)	Open Science Framework (10.17605/OSF.IO/2WURJ)

The total sample sizes for the original surveys were 78,623 for boys and 74,372 for girls (a total of 152,995 names). The average annual sample sizes were 5242 for boys and 4958 for girls.

### Hiragana name

Although most Japanese names are written using Chinese characters, other types of characters can be used for names. In fact, within the top 10 most common writings, hiragana names were included. The numbers of variations and hiragana names within the top 10 most common writings are summarized in Table S2 (see Table 1). There were no boys’ names written using hiragana characters within the top 10 most common writings. In contrast, for girls’ names, in most years, one or two hiragana names were included in the rankings. The names were “さくら” (Sakura; meaning a cherry blossom/tree) and “ひなた” (Hinata; meaning a sunny place).

### Advantages

The present data have advantages: (1) they include both writings and readings of baby names, (2) they were collected under the same conditions over 15 years, (3) their sample sizes are relatively large, and (4) they are open to the public. These are explained in more detail in Supplementary material (see Table 1).

## Limitations

### The samples are not highly representative

It would be difficult to state that the samples are highly representative of names that were given to babies in a given year. The present data are based on the original data collected by Meiji Yasuda. It may be true that the original data are not representative of the population of interest. Moreover, the present data are from the lists of readings of the top 10 most common writings, not all names that were collected in the original surveys.

Data that are highly representative of a nation must be provided by the national government (e.g., identity card, resident registration). For example, in the U.S., the government (the Social Security Administration) publishes data including almost the entire population (approximately four million) every year [13]. In Japan, such data are unfortunately unavailable, at least at present. Thus, conclusions should be drawn with this constraint in mind.

### The sample sizes are not very large

Although the sample sizes are sufficiently large to conduct various empirical analyses, as explained above, it is difficult to state that the sizes are large, considering that approximately one million babies received names every year in Japan. This limitation is also caused by the difficulty of collecting private information and the unavailability of governmental data.

## Data Availability

The data generated and/or analysed during the current study can be freely and openly accessed on Open Science Framework under 10.17605/OSF.IO/2WURJ. Please see Table 1 and reference [12] for details and links to the data.
